# Ru/Me-BIPAM-Catalyzed Asymmetric Addition of Arylboronic Acids to Aliphatic Aldehydes and α-Ketoesters

**DOI:** 10.3390/molecules16065020

**Published:** 2011-06-17

**Authors:** Yasunori Yamamoto, Tomohiko Shirai, Momoko Watanabe, Kazunori Kurihara, Norio Miyaura

**Affiliations:** Division of Chemical Process Engineering, Graduate School of Engineering, Hokkaido University, kita 13, Nishi 8, Kita-ku, Sapporo 060-8628, Japan

**Keywords:** asymmetric catalyst, bidentate phosphoramidite ligand, ruthenium catalyzed arylation

## Abstract

A ruthenium-catalyzed asymmetric arylation of aliphatic aldehydes and α-ketoesters with arylboronic acids has been developed, giving chiral alkyl(aryl)methanols and α-hydroxy esters in good yields. The use of a chiral bidentate phosphoramidite ligand (Me-BIPAM) achieved excellent enantioselectivities.

## 1. Introduction

**Scheme 1:**
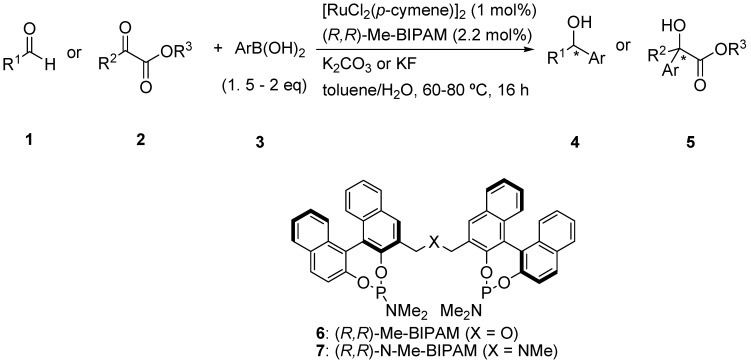
Arylation of aliphatic aldehydes and α-ketoesters.

Transmetalation between organoboronic reagents and transition metals is a fundamental process involved in many metal-catalyzed C-C bond-forming reactions [1,2]. In this field, we reported a new catalytic cycle starting from transmetalation to give an organorhodium(I), -palladium(II) or -ruthenium(II) intermediate for 1,4-addition of organoboronic acids to electron-deficient alkenes and arylation of the carbon-heteroatom double bond of aldehydes and N-sulfonylimines [3,4,5]. We have developed new bidentate chiral phosphoramidites [Me-BIPAM (**6**), N-Me-BIPAM (**7**)] based on linked-BINOL for enantioselective 1,4-addition of arylboronic acids to enones [6,7], arylation of aldimines [8] and hydrogenation of α-dehydroamino esters [9] with rhodium catalysts. These ligands were also found to be highly efficient for ruthenium-catalyzed enantioselective arylation of aromatic aldehydes [10]. Herein, we report arylation of aliphatic aldehydes **1** and α-ketoesters **2** with arylboronic acids **3** catalyzed by a chiral ruthenium complex, generated *in situ* from [RuCl_2_(*p*-cymene)]_2_ and (*R,R*)-Me-BIPAM (**6**) (Scheme 1).

## 2. Results and Discussion

The arylation of carbonyl compounds with organolithium [11,12], organomagnesium [13,14,15] and organozinc [16,17,18,19,20,21] reagents are the traditional ways to access alkyl(aryl)methanol and α-hydroxy-esters, but there has been recent interest in the transition-metal-catalyzed arylation using tin [22] and boron [23,24,25,26,27] compounds. Since the corresponding rhodium complexes were inefficient, we previously developed a highly enantioselective arylation of aldehydes with boronic acids by using ruthenium catalyst [10]. In our continuing program to expand the utility of the ruthenium/Me-bipam catalyst, we planned to develop an enantioselective addition of arylboronic acids to aliphatic aldehydes. [RuCl_2_(*p*-cymene)]/Me-bipam (2 mol%) catalyzed the addition of arylboronic acids to representative aliphatic aldehydes in high yields in the presence of one equivalent of K_2_CO_3_ at 60 °C in toluene/H_2_O (10:1). A variety of aliphatic aldehydes underwent the arylation reaction (Table 1). Not only linear aliphatic aldehydes but also branched ones participated in the arylation reaction. Most reactions took place smoothly in toluene/H_2_O (10/1), but toluene/H_2_O (5/1) was a better solvent for the slow addition (Table 1, entries 1, 6, 10, 11, 17-19).

Next, we employed Ru/Me-BIPAM as the catalyst for the addition reaction of arylboronic acids to α-ketoesters, could yield useful α-hydroxy-esters with α-quaternary carbon centers. The rhodium(I)/(*S*)-Ship complex developed by Zhou and co-workers was the most promising catalyst, achieving 80-93% ee for 2-oxo-2-arylacetate and 2-oxo-4-phenyl-3-butenoate [29]. Several bases were screened for the reactions involving a [RuCl_2_(*p*-cymene)]_2_/2Me-bipam catalyst (Table 2). 

**Table 1:** Arylation of aliphatic aldehydes *^a^*. 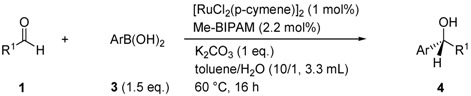

Entry	R^1^ =	Ar =	Yield (%)	ee (%) (abs)
1 *^b^*	*n*-C_2_H_5_ (**1a**)	Ph (**3a**)	63 (**4aa**)	91 (*R*)
2	*n*-C_4_H_9_ (**1b**)	Ph (**3a**)	93 (**4ba**)	94 (*R*)
3	*n*-C_4_H_9_ (**1b**)	2-naphthyl (**3b**)	98 (**4bb**)	93 (*R*)
4	*n*-C_4_H_9_ (**1b**)	4-MeC_6_H_4_ (**3c**)	85 (**4bc**)	92 (*R*)
5	*n*-C_4_H_9_ (**1b**)	4-MeOC_6_H_4_ (**3d**)	93 (**4bd**)	92 (*R*)
6 *^b,c^*	*n*-C_4_H_9_ (**1b**)	4-ClC_6_H_4_ (**3e**)	90 (**4be**)	87 (*R*)
7	*n*-C_4_H_9_ (**1b**)	4-FC_6_H_4_ (**3f**)	69 (**4bf**)	91 (*R*)
8	*n*-C_4_H_9_ (**1b**)	3-MeOC_6_H_4_ (**3h**)	62 (**4bh**)	90 (*R*)
9	*n*-C_4_H_9_ (**1b**)	3-ClC_6_H_4_ (**3i**)	63 (**4bi**)	90 (+)
10 *^b,d^*	*n*-C_4_H_9_ (**1b**)	3-F-4-MeOC_6_H_3_ (**3k**)	65 (**4bk**)	87 (+)
11 *^b,c^*	*n*-C_4_H_9_ (**1b**)	3,4-(CH_2_O_2_)C_6_H_3_ (**3l**)	58 (**4bl**)	99 (*R*)
12	*n*-C_5_H_11_ (**1c**)	Ph (**3a**)	91 (**4ca**)	94 (*R*)
13	*n*-C_6_H_13_ (**1d**)	Ph (**3a**)	93 (**4da**)	93 (*R*)
14 *^c^*	*n*-C_8_H_17_ (**1e**)	Ph (**3a**)	87 (**4ea**)	92 (*R*)
15	PhCH_2_CH_2_ (**1f**)	Ph (**3a**)	99 (**4fa**)	92 (*R*)
16	*cyclo*-C_6_H_11_ (**1g**)	Ph (**3a**)	78 (**4ga**)	94 (*R*)
17 *^b^*	*i*-Pr (**1h**)	Ph (**3a**)	67 (**4ha**)	96 (*R*)
18 *^b,c^*	(C_2_H_5_)_2_CH (**1j**)	Ph (**3a**)	54 (**4ja**)	91 (*R*)
19 *^b,c,e^*	*t*-Bu (**1k**)	Ph (**3a**)	40 (**4ka**)	99 (*R*)

*^a^* Reaction conditions: A mixture of aldehyde (0.5 mmol), ArB(OH)_2_ (0.75 mmol), K_2_CO_3_ (0.5 mmol), [RuCl_2_(*p*-cymen)]_2_ (1 mol%) and (*R,R*)-Me-BIPAM (2.2 mol%) in toluene (3 mL) and H_2_O (0.3 mL) was stirred at 60 °C for 16 h. *^b^* toluene/H_2_O (5/1) was used. *^c^* at 80 °C. *^d^* KOH was used. *^e^* K_3_PO_4_ was used.

**Table 2:** Reaction conditions *^a^*. 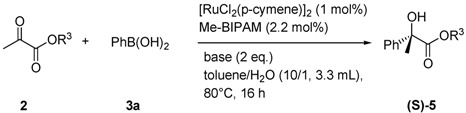

Entry	Base	R^3^ =	Yield (%)	ee (%) (abs)
1 *^b^*	K_2_CO_3_	Et	40	93
2 *^b^*	K_3_PO_4_	Et	trace	ND
3 *^b^*	CsF	Et	40	93
4 *^b^*	KF	Et	71	95
5	KF	Et	78	94
**6**	**KF**	***i*-Pr (2a)**	**85**	**93 (*S*)**
7	KF	*t*-Bu	87	90
8 *^c^*	KF	*t*-Bu	72	70

*^a^* Reaction conditions: A mixture of alkyl pyruvate (0.5 mmol), PhB(OH)_2_ (1.0 mmol), base (1.0 mmol), [RuCl_2_(*p*-cymen)]_2_ (1 mol%) and (*R,R*)-Me-BIPAM (2.2 mol%) in toluene (3 mL) and H_2_O (0.3 mL) was stirred at 80 °C for 16 h. *^b^* at 50 °C. *^c^* (*R,R*)-N-Me-BIPAM was used.

K_2_CO_3_, K_3_PO_4_ or CsF resulted in lower yields (Table 2, entries 1-3). The highest efficiency with regard to the reaction was observed when KF was used for the arylation of isopropyl pyruvate with phenylboronic acid at 80 °C (Table 2, entry 6). The yield of the product was dependent on the bulkiness of the ester moiety of the substrate (Table 2, entries 5-7), and the best results were obtained with isopropyl ester as the substrate. Among chiral ligands screened, *N*-Me-bipam (**7**) achieved a 70% ee (entry 8). Substrate generality was then investigated under the optimized reaction conditions (Table 3). High ee values were obtained with methyl, ethyl, and phenyl-substituted ketoesters. Representative *meta*- and *para*-substituted arylboronic acids with electron-donating or electron-withdrawing substituents afforded good yields of tertiary α-hydroxy-esters with high enantioselectivities. (*R,R*)-Me-bipam has given the products **4** and **5** by the same enantioselection. To elucidate the enantioselection in the mechanism, the characterization of the catalyst and the intermediate are in progress.

**Table 3:** Arylation of α-ketoesters *^a^*. 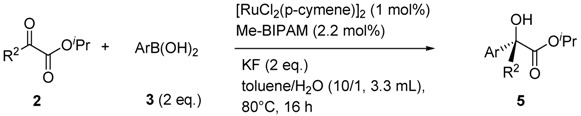

Entry	R^2^ =	Ar =	Yield (%)	ee (%) (abs)
1	Me (**2a**)	Ph (**3a**)	85 (**5aa**)	93 (*S*)
2	Me (**2a**)	4-MeC_6_H_4_ (**3c**)	84 (**5ac**)	89
3	Me (**2a**)	4-MeOC_6_H_4_ (**3d**)	84 (**5ad**)	91
4	Me (**2a**)	4-FC_6_H_4_ (**3f**)	85 (**5af**)	93
5	Me (**2a**)	4-CF_3_C_6_H_4_ (**3g**)	64 (**5ag**)	92
6	Me (**2a**)	3-MeOC_6_H_4_ (**3h**)	73 (**5ah**)	92
7	Me (**2a**)	3-FC_6_H_4_ (**3j**)	71 (**5aj**)	90
8	Me (**2a**)	3-F-4-MeOC_6_H_3_ (**3k**)	81 (**5ak**)	87
9	Et (**2b**)	Ph (**3a**)	88 (**5ba**)	95
10	Et (**2b**)	4-MeC_6_H_4_ (**3c**)	90 (**5bc**)	91
11	Et (**2b**)	4-FC_6_H_4_ (**3f**)	90 (**5bf**)	93
12	Et (**2b**)	3-MeOC_6_H_4_ (**3h**)	88 (**5bh**)	91
13	*i-*Pr (**2c**)	Ph (**3a**)	41 (**5ca**)	94
14	*i-*Pr (**2c**)	4-MeOC_6_H_4_ (**3d**)	42 (**5cd**)	90
15	Ph (**2d**)	4-MeC_6_H_4_ (**3c**)	82 (**5dc**)	92
16	Ph (**2d**)	4-MeOC_6_H_4_ (**3d**)	95 (**5dd**)	86
17	Ph (**2d**)	4-ClC_6_H_4_ (**3e**)	90 (**5de**)	91
18	Ph (**2d**)	4-FC_6_H_4_ (**3f**)	90 (**5df**)	94
19	Ph (**2d**)	3-MeOC_6_H_4_ (**3h**)	79 (**5dh**)	92
20	4-FC_6_H_4_ (**2e**)	3-ClC_6_H_4_ (**3i**)	67 (**5ei**)	90

^a^ Reaction conditions: A mixture of α-ketoester (0.5 mmol), ArB(OH)_2_ (1.0 mmol), KF (1.0 mmol), [RuCl_2_(*p*-cymen)]_2_ (1 mol%) and (*R,R*)-Me-BIPAM (2.2 mol%) in toluene (3 mL) and H_2_O (0.3 mL) was stirred at 80 °C for 16 h.

## 3. Experimental Section

### 3.1. General

^1^H–NMR spectra were recorded on a JEOL ECX-400 (400 MHz) in CDCl_3_ with tetramethylsilane as an internal standard. Chemical shifts are reported in part per million (ppm), and signal are expressed as singlet (s), doublet (d), triplet (t), quartet (q), multiplet (m), and broad (br). ^13^C-NMR spectra were recorded on a JEOL ECX-400 (100 MHz) in CDCl_3_ (δ_C_ = 77.0) with tetramethylsilane as an internal standard. Chemical shifts are reported in part per million (ppm). HPLC analysis was directly performed with chiral stationary phase column, Chiralpak AD-H, IB or Chiralcel OD-H, OB-H purchased from DAICEL Co., Ltd. High resolution mass spectra (HRMS) were recorded on a JEOL JMS 700TZ mass spectrometer at the Center *for Instrumental Analysis*, Hokkaido University. Optical rotations were measured on a HORIBA SEPA-300 digital polarimeter. Kanto Chemical silica gel 60N (particle size 0.063-0.210 mm) was used for flash column chromatography. RuCl_3_·xH_2_O were purchesed from Strem Chemical, Inc. [RuCl_2_(p-cymene)]_2_ [28], BIPAM ligands (Me-BIPAM, N-Me-BIPAM) were prepared according to our previous procedure [7,8]. Me-BIPAM was commercially available from Wako Pure Chemical Industries, Ltd. 

### 3.2. General Procedure for Arylation of Aliphatic Aldehydes (Table 1)

A flask was charged with [RuCl_2_(*p*-cymene)]_2_ (0.005 mmol, 1 mol%) and (*R,R*)-Me-bipam (0.011 mmol, 2.2 mol%) under a nitrogen atmosphere. Toluene (3.0 mL) was added to the flask and the mixture was then stirred at room temperature for 30 min to prepare the catalyst. Pentanal (**1b**, 0.5 mmol), phenylboronic acid (**3a**, 0.75 mmol), K_2_CO_3_ (0.5 mmol), and H_2_O (0.3 mL) were then added to this catalyst solution. The reaction mixture was stirred at 60 °C for 16 h, at which time the crude reaction mixture extracted using ethyl acetate, washed with saturated NH_4_Cl and brine, and dried over MgSO_4_. Chromatography of the crude reaction mixture on silica gel gave *(R)-1-phenyl-1-pentanol* (**4ba**) [29] in 93% yield; [α]_D_^23^ = 33.8 (c 0.80, C_6_H_6_), 94% ee [HPLC conditions: Chiralcel OD, hexane/2-propanol = 99/1, flow = 0.85 mL min^−1^, wavelength = 254 nm, t_major_ = 22.7 and t_minor_ = 26.6 min]; ^1^H-NMR (400 MHz, CDCl_3_): δ = 7.34-7.25 (m, 5H), 4.65 (t, *J* = 6.8 Hz, 1H), 1.86-1.70 (m, 3H), 1.39-1.25 (m, 4H), 0.88 (t, *J* = 7.0 Hz, 3H); HRMS *m/z*; calcd. For C_11_H_16_O: 164.1201; found 164.1203.

*(R)-1-Phenyl-1-propanol* (**4aa**) [29,30]: [α]_D_^21^ = 43.4 (c 0.87, CHCl_3_) 91% ee [HPLC conditions: Chiralpak OD, hexane/2-propanol = 99/1, flow = 0.8 mL min^−1^, wavelength = 254 nm, t_major_ = 26.0 and t_minor_ = 33.0 min]; ^1^H-NMR (400 MHz, CDCl_3_): δ = 7.34-7.25 (m, 5H), 4.60 (t, *J* = 6.6 Hz, 1H), 1.88-1.71 (m, 3H), 0.91 (t, *J* = 7.5 Hz, 3H); HRMS *m/z*; calcd. for C_9_H_12_O: 136.08881; found 136.08881.

*(R)-1-(-2-Naphthyl)-1-pentanol* (**4bb**) [31]: [α]_D_^19^ = 33.8 (c 1.52, CHCl_3_), 93% ee [HPLC conditions: Chiralcel OD, hexane/2-propanol = 50/1, flow = 1.0 mL min^−1^, wavelength = 254 nm, t_minor_ = 27.0 and t_major_ = 29.6 min]; ^1^H-NMR (400 MHz, CDCl_3_): δ = 7.87-7.77 (m, 4H), 7.51-7.45 (m, 3H), 4.85 (t, *J* = 6.6 Hz, 1H), 1.94-1.79 (m, 3H), 1.46-1.26 (m, 4H), 0.89 (t, *J* = 7.0 Hz, 3H).

*(R)-1-(4-Tolyl)-1-pentanol* (**4bc**) [29,32]: [α]_D_^22^ = 29.4 (c 0.80, C_6_H_6_), 92% ee [HPLC conditions: Chiralcel OJ, hexane/2-propanol = 200/1, flow = 1.0 mL min^−1^, wavelength = 254 nm, t_major_ = 20.4 and t_minor_ = 22.6 min]; ^1^H-NMR (400 MHz, CDCl_3_): δ = 7.26-7.15 (m, 4H), 4.63 (t, *J* = 6.2 Hz, 1H), 2.34 (s, 3H), 1.85-1.64 (m, 3H), 1.42-1.20 (m, 4H), 0.88 (t, *J* = 7.0 Hz, 3H).

*(R)-1-(4-Methoxyphenyl)-1-pentanol* (**4bd**) [33]: [α]_D_^19^ = 26.9 (c 0.37, CHCl_3_), 92% ee [HPLC conditions: Chiralcel OD, hexane/2-propanol = 99/1, flow = 0.8 mL min^−1^, wavelength = 254 nm, t_major_ = 37.4 and t_minor_ = 41.0 min]; ^1^H-NMR (400 MHz, CDCl_3_): δ = 7.27 (d, *J* = 8.0 Hz,2H ), 6.89 (d, *J* = 8.8 Hz,2H), 4.61 (t, *J* = 6.8 Hz, 1H), 3.81 (s, 3H), 1.85-1.64 (m, 2H), 1.41-1.20 (m, 4H), 0.88 (t, *J* = 7.4 Hz, 3H); HRMS *m/z*; calcd. for C_12_H_18_O_2_: 194.13068; found 194.13084. 

*(R)-1-(4-Chlorophenyl)-1-pentanol* (**4be**) [29]: [α]_D_^22^ = 18.3 (c 0.60, C_6_H_6_), 87% ee [HPLC conditions: Chiralcel OD, hexane/2-propanol = 99/1, flow = 0.8 mL min^−1^, wavelength = 230 nm, t_minor_ = 24.5 and t_major_ = 27.0 min]; ^1^H-NMR (400 MHz, CDCl_3_): δ = 7.34-7.29 (m, 4H), 4.66 (t, *J* = 7.2 Hz, 1H), 1.82-1.56 (m, 3H), 1.40-1.26 (m, 4H), 0.88 (t, *J* = 6.2 Hz, 3H); HRMS *m/z*; calcd. for C_11_H_15_C1O: 198.08114; found 198.08132. 

*(R)-1-(4-Fluorophenyl)-1-pentanol* (**4bf**) [34]: [α]_D_^21^ = 40.5 (c 0.50, CHCl_3_), 91% ee [HPLC conditions: Chiralpak AD-H, hexane/2-propanol = 99/1, flow = 0.8 mL min^−1^, wavelength = 230 nm, t_minor_= 27.3 and t_major_ = 31.5 min]; ^1^H-NMR (400 MHz, CDCl_3_): δ = 7.34-7.26 (m, 3H), 7.06-7.00 (t, *J* = 8.5 Hz, 2H), 4.66 (t, *J* = 6.2 Hz, 1H), 1.78-1.61 (m, 3H), 1.35-1.26 (m, 4H), 0.87 (t, *J* = 6.2 Hz, 3H); ^13^C-NMR (100 MHz, CDCl_3_): δ = 162.2 (d, *J* = 245 Hz), 140.7 (d, *J* = 2.86 Hz), 127.6 (d, *J* = 7.63 Hz), 115.3 (d, *J* = 20.98 Hz), 74.1, 39.0, 28.0, 22.7, 14.1; HRMS *m/z*; calcd. for C_11_H_15_FO: 182.11069; found 182.11040. 

*(R)-1-(3-Methoxyphenyl)-1-pentanol* (**4bh**) [35]: [α]_D_^20^ = 30.2 (c 0.90, THF), 90% ee [HPLC conditions: Chiralcel OD, hexane/2-propanol = 99/1, flow = 0.7 mL min^−1^, wavelength = 254 nm, t_major_ = 58.3 and t_minor_ = 67.9 min]; ^1^H-NMR (400 MHz, CDCl_3_): δ= 7.26 (s, 1H), 7.14-7.02 (m, 2H), 6.92 (t, *J* = 8.4 Hz, 1H), 4.64 (t, *J* = 6.6 Hz, 1H), 3.81 (s, 3H), 1.83-1.63 (m, 3H), 1.39-1.19 (m, 4H), 0.88 (t, *J* = 7.0 Hz, 3H); HRMS m/z; calcd. for C_12_H_18_O_2_: 194.13068; found 194.13040. 

*1-(3-Chlorophenyl)-1-pentanol* (**4bi**) [32]: [α]_D_^20^ = 24.0 (c 0.39, CHCl_3_), 90% ee [HPLC conditions: Chiralcel OD, hexane/2-propanol = 99/1, flow = 0.8 mL min^−1^, wavelength = 230 nm, t_minor_ = 23.1 and t_major_ = 25.4 min]; ^1^H-NMR (400 MHz, CDCl_3_): δ = 7.40-7.18 (m, 4H), 4.64 (t, *J* = 6.6 Hz, 1H), 1.83-1.61 (m, 2H), 1.41-1.20 (m, 4H), 0.88 (t, *J* = 7.0 Hz, 3H); HRMS *m/z*; calcd. for C_11_H_15_ClO: 198.08114; found 198.08097. 

*1-(3-Fluoro-4-methoxyphenyl)-1-pentanol* (**4bk**): [α]_D_^20^ = 23.6 (c 0.33, CHCl_3_), 87% ee [HPLC conditions: Chiralcel OD, hexane/2-propanol = 99/1, flow = 0.75 mL min^−1^, wavelength = 230 nm, t_major_ = 37.0 and t_minor_ = 40.7 min]; ^1^H-NMR (400 MHz, CDCl_3_): δ = 6.94-6.89 (m, 2H), 6.84-6.79 (m, 1H), 4.65 (t, *J* = 6.6 Hz, 1H), 3.82 (s, 3H), 1.84-1.64 (m, 3H), 1.42-1.22 (m, 4H), 0.89 (t,*J* = 7.4 Hz, 3H); ^13^C-NMR (100 MHz, CDCl_3_): δ = 152.4 (d, *J* = 246 Hz), 146.9 (d, *J* = 10.49 Hz), 138.2 (d, *J* = 4.77 Hz), 121.7 (d, *J* = 3.81 Hz), 113.8 (d, *J* = 18.12 Hz), 113.2, 73.9, 56.4, 38.8, 28.0, 22.7, 14.1; HRMS *m/z*; calcd. for C_12_H_17_FO_2_: 212.12126; found 212.12104. 

*(R)-1-(5-Benzo[d][1,3]dioxolyl)-1-pentanol* (**4bl**) [36]: [α]_D_^20^ = 62.4 (c 0.48, CHCl_3_), 99% ee [HPLC conditions: Chiralcel OD, hexane/2-propanol = 99/1, flow = 0.8 mL min^−1^, wavelength = 254 nm, t_minor_= 39.9 (S) and t_major_ = 44.8 min]; ^1^H-NMR (400 MHz, CDCl_3_): δ = 6.87 (s, 1H), 6.78 (s, 2H), 5.95 (s, 2H), 4.58 (t, *J* = 7.2 Hz, 1H), 1.83-1.59 (m, 3H), 1,40-1.18 (m, 4H), 0.88 (t, *J* = 7.0 Hz, 3H).

*(R)-1-Phenyl-1-hexanol* (**4ca**) [37]: [α]_D_^23^ = 37.5 (c 0.82, CHCl_3_), 94% ee [HPLC conditions: Chiralcel OD, hexane/2-propanol = 99/1, flow = 0.9 mL min^−1^, wavelength = 254 nm, t_major_ = 19.2 and t_minor_ = 22.4 (S) min]; ^1^H-NMR (400 MHz, CDCl_3_): δ = 7.34-7.25 (m, 5H), 4.66 (t, *J* = 6.8 Hz, 1H), 1.87-1.61 (m, 3H), 1.42-1.22 (m, 6H), 0.88 (t, *J* = 7.0 Hz, 3H); HRMS *m/z*; calcd. for C_12_H_18_O: 178.1358; found 178.1353.

*(R)-1-Phenyl-1-heptanol* (**4da**) [38,39]: [α]_D_^23^ = 31.2 (c 0.85, CHCl_3_ ), 93% ee [HPLC conditions: Chiralcel OD, hexane/2-propanol = 99/1, flow = 0.9 mL min^−1^, wavelength = 254 nm, t_major_ = 19.9 and t_minor_ = 22.9 min]; ^1^H-NMR (400 MHz, CDCl_3_): δ = 7.34-7.25 (m, 6H), 4.66 (t, *J* = 6.6 Hz, 1H), 1.84-1.56 (m, 2H), 1.40-1.25 (m, 8H), 1.42-1.23 (m, 12H), 0.86 (t, *J* = 6.6 Hz, 3H); HRMS *m/z*; calcd. for C_13_H_20_O: 192.1514; found 192.1511.

*(R)-1-Phenyl-1-nonanol* (**4ea**) [40,41]: [α]_D_^19^ = 27.3 (c 1.42, CHCl_3_ ), 92% ee [HPLC conditions: Chiralcel OD, hexane/2-propanol = 99/1, flow = 0.7 mL min^−1^, wavelength = 254 nm, t_major_ = 25.0 and t_minor_ = 31.8 min]; ^1^H-NMR (400 MHz, CDCl_3_): δ = 7.34-7.25 (m, 5H), 4.66 (t, *J* = 6.1 Hz, 1H), 1.93-1.65 (m, 3H), 1.42-1.19 (m, 12H), 0.87 (t, *J* = 6.6 Hz, 3H); HRMS *m/z*; calcd. for C_15_H_24_O: 220.1827; found 220.1822. 

*(R)-1,3-Diphenyl-1-propanol* (**4fa**) [42]: [α]_D_^20^ = 15.6 (c 0.85, CH_2_Cl_2_), 92% ee [HPLC conditions: Chiralcel OD, hexane/2-propanol = 95/5, flow = 0.7 mL min^−1^, wavelength = 254 nm, t_minor_ = 28.2 and t_major_ = 33.7 min]; ^1^H-NMR (400 MHz, CDCl_3_): δ = 7.38-7.14 (m, 10H), 4.68 (t, *J* = 6.6 Hz, 1H), 2.77-2.65 (m, 2H), 2.15-2.02 (m, 2H), 1.92 (s, 1H); HRMS *m/z*; calcd. for C_15_H_16_O: 212.1201; found 212.1197. 

*(R)-Cyclohexyl(phenyl)methanol* (**4ga**) [33]: [α]_D_^20^ = 39.5 (c 0.23, CHCl_3_), 94% ee [HPLC conditions: Chiralcel OD, hexane/2-propanol = 99/1, flow = 0.4 mL min^−1^, wavelength = 254 nm, t_minor_ = 45.2 and t_major_ = 48.8 min]; ^1^H-NMR (400 MHz, CDCl_3_): δ = 7.35-7.24 (m, 5H), 4.35 (d, *J* = 7.3 Hz, 1H), 2.03-1.60 (m, 6H), 1.38-0.90 (m, 6H); HRMS *m/z*; calcd. for C_13_H_18_O: 190.1358; found 190.1358. 

*(R)-2-Methyl-1-phenyl-1-propanol* (**4ha**) [33]: [α]_D_^19^ = 11.3 (c 0.42, CHCl_3_), 96% ee [HPLC conditions: Chiralpak AD-H, hexane/2-propanol = 99/1, flow = 1.0 mL min^−1^, wavelength = 254 nm, t_major_ = 17.6 and t_minor_ = 18.8 min]; ^1^H-NMR (400 MHz, CDCl_3_): δ = 7.35-7.28 (m, 5H), 4.35 (d, *J* = 6.8 Hz, 1H), 2.00-1.89 (m, 1H), 1.82 (broad s, 1H), 1.00 (d, *J* = 6.8 Hz, 3H), 0.79 (d,*J* = 6.8 Hz, 3H); HRMS *m/z*; calcd. for C_10_H_14_O: 150.1045; found 150.1043. 

*(R)-2-Ethyl-1-phenyl-1-butanol* (**4ja**) [43]: [α]_D_^20^ = −10.6 (c 0.35, CHCl_3_), 91% ee [HPLC conditions: Chiralcel OD, hexane/2-propanol = 99/1, flow = 0.5 mL min^−1^, wavelength = 254 nm, t_major_ = 29.2 and t_minor_ = 44.5 min]; ^1^H-NMR (400 MHz, CDCl_3_): δ = 7.36-7.24 (m, 5H), 4.63 (d, *J* = 6.3 Hz, 1H), 1.77 (broad s, 1H), 1.60-1.40 (m, 2H), 0.90-0.82 (m, 6H); HRMS *m/z*; calcd. for C_12_H_18_O: 178.1358; found 178.1354. 

*(R)-2,2-Dimethyl-1-phenyl-1-propanol* (**4ka**) [33]: [α]_D_^20^ = 19.2 (c 0.48, CHCl_3_), 99% ee [HPLC conditions: Chiralpak OD, hexane/2-propanol = 98/2, flow = 0.9 mL min^−1^, wavelength = 254 nm, t_minor_ = 7.9 and t_major_ = 11.7 min]; ^1^H-NMR (400 MHz, CDCl_3_): δ = 7.32-7.26 (m, 5H), 4.40 (s, 1H), 0.93 (s, 9H).

### 3.3. General Procedure for Arylation of α-Ketoesters (Table 3)

A flask was charged with [RuCl_2_(*p*-cymene)]_2_ (0.005 mmol, 1 mol%) and (*R,R*)-Me-bipam (0.011 mmol, 2.2 mol%) under a nitrogen atmosphere. Toluene (3.0 mL) was added to the flask and the mixture was then stirred at room temperature for 30 min to prepare the catalyst. Isopropyl pyruvate (**2a**, 0.5 mmol), phenylboronic acid (**3a**, 0.75 mmol), KF (1.0 mmol), and H_2_O (0.3 mL) were then added to this catalyst solution. The reaction mixture was stirred at 80 °C for 16 h, at which time the crude reaction mixture extracted using ethyl acetate, washed with saturated NH_4_Cl and brine, and dried over MgSO_4_. Chromatography of the crude reaction mixture on silica gel gave *(S)-isopropyl 2-hydroxy-2-phenylpropanoate* (**5aa**) in 85% yield [44,45,46]. [α]_D_^22^ = +40.00 (c 4.2, CHCl_3_), 93% ee [HPLC conditions: Chiralcel OJ-H column, hexane/2-propanol = 98/2, flow = 1.0 mL/min, wavelength = 230 nm, t_major_ = 8.0 min and t_minor_ = 16.4 min]; ^1^H-NMR (400 MHz, CDCl_3_) δ = 7.54-7.57 (m, 2H), 7.23-7.36 (m, 4H), 5.05 (sep, *J* = 6.4 Hz, 1H), 3.85 (s, 1H), 1.75 (s, 3H), 1.28 (d, *J* = 6.4 Hz, 3H), 1.17 (d, *J* = 6.0 Hz, 3H); ^13^C-NMR (100 MHz, CDCl_3_) δ = 175.3, 143.1, 128.3, 127.7, 125.2, 75.7, 70.4, 26.7, 21.8, 21.5; HRMS *m/z*; calcd. for C_12_H_16_O_3_Na: 231.09917; found 231.09919.

*Isopropyl* *2-hydroxy-2-(4-tolyl)propanoate* (**5ac**): [α]_D_^22^ = +34.40 (c 4.8, CHCl_3_), 89% ee [HPLC conditions: Chiralcel OJ-H column, hexane/2-propanol = 98/2, flow = 1.0 mL/min, wavelength = 230 nm, t_major_ = 7.7 min and t_mainor_ = 14.8 min]; ^1^H-NMR (400 MHz, CDCl_3_) δ = 7.43 (d, *J* = 8.2 Hz, 2H), 7.14 (d, *J* = 8.2 Hz, 2H), 5.04 (sep, *J* = 6.4 Hz, 1H), 3.78 (s, 1H), 2.33 (s, 3H), 1.73 (s, 3H), 1.27 (d, 3H, *J* = 6.5 Hz), 1.18 (d, *J* = 6.4 Hz, 3H); ^13^C-NMR (100 MHz, CDCl_3_) δ = 175.4, 140.2, 137.4, 129.0, 125.1, 75.5, 70.3, 26.7, 21.8, 21.5, 21.1; HRMS *m/z*; calcd. for C_13_H_18_O_3_Na: 245.11482; found 245.11494.

*Isopropyl* *2-hydroxy-2-(4-methoxyphenyl)propanoate* (**5ad**): [α]_D_^22^ = +37.85 (c 5.1, CHCl_3_), 91% ee [HPLC conditions: Chiralcel OJ-H column, hexane/2-propanol = 98/2, flow = 1.0 mL/min, wavelength = 230 nm, t_major_ = 14.1 min and t_minor_ = 31.9 min]; ^1^H-NMR (400 MHz, CDCl_3_) δ = 7.45 (d, *J* = 8.7 Hz, 2H), 6.85 (d, *J* = 8.7 Hz, 2H), 5.03 (sep, *J* = 6.4 Hz, 1H), 3.79 (s, 4H), 1.72 (s, 3H), 1.27 (d, *J* = 6.4 Hz, 3H), 1.16 (d, *J* = 6.0 Hz, 3H); ^13^C-NMR (100 MHz, CDCl_3_) δ = 175.5, 159.1, 135.2, 126.5, 113.6, 75.3, 70.3, 55.3, 26.7, 21.8, 21.5; HRMS *m/z*; calcd. for C_13_H_18_O_4_Na: 261.10973; found 261.10988.

*Isopropyl* *2-hydroxy-2-(4-fluorophenyl)propanoate* (**5af**): [α]_D_^22^ = +39.19 (c 5.0, CHCl_3_), 93% ee [HPLC conditions: Chiralcel OJ-H column, hexane/2-propanol = 98/2, flow = 1.0 mL/min, wavelength = 230 nm, t_major_ = 7.2 min and t_minor_ = 10.0 min]; ^1^H-NMR (400 MHz, CDCl_3_) δ = 7.50-7.55 (m, 2H), 6.98-7.03 (m, 2H), 5.04 (sep, *J* = 6.4 Hz, 1H), 3.87 (d, *J* = 0.9 Hz, 1H), 1.73 (s, 3H), 1.27 (d, *J* = 6.4 Hz, 3H), 1.16 (d, *J* = 6.0 Hz, 3H); ^13^C-NMR (100 MHz, CDCl_3_) δ = 175.1, 162.4 (d, *J* = 246 Hz), 138.8 (d, *J* = 2.86 Hz), 127.2 (d, *J* = 8.58 Hz), 115.1 (d, *J* = 21.93 Hz), 75.2, 70.5, 26.9, 21.7, 21.5; HRMS *m/z*; calcd. for C_12_H_15_O_3_FNa: 249.08974; found 249.08998.

*Isopropyl* *2-hydroxy-2-(4-trifluoromethylphenyl)propanoate* (**5ag**): [α]_D_^22^ = +30.04 (c 4.2, CHCl_3_), 92% ee [HPLC conditions: Chiralcel OJ-H column, hexane/2-propanol = 98/2, flow = 1.0 mL/min, wavelength = 230 nm, t_major_ = 8.6 min and t_minor_ = 11.6 min]; ^1^H-NMR (400 MHz, CDCl_3_) δ = 7.70 (d, *J* = 8.2 Hz, 2H), 7.59 (d, *J* = 8.2 Hz, 2H), 5.05 (sep, *J* = 6.4 Hz, 1H), 3.91 (s, 1H), 1.76 (s, 3H), 1.29 (d, *J* = 6.4 Hz, 3H), 1.18 (d, *J* = 6.4 Hz, 3H); ^13^C-NMR (100 MHz, CDCl_3_) δ = 174.6, 146.9, 130.0 (q, *J* = 32.4 Hz), 125.8, 125.5, 125.2 (q, *J* = 3.81 Hz), 122.8, 75.5, 70.9, 27.0, 21.7, 21.5; HRMS *m/z*; calcd. for C_13_H_15_O_3_F_3_Na: 299.08655; found 299.08701.

*Isopropyl* *2-hydroxy-2-(3-methoxyphenyl)propanoate* (**5ah**): [α]_D_^22^ = +26.54 (c 5.1, CHCl_3_), 92% ee [HPLC conditions: Chiralcel OJ-H column, hexane/2-propanol = 98/2, flow = 1.0 mL/min, wavelength = 230 nm, t_major_ = 10.4 min and t_mainor_ = 20.5 min]; ^1^H-NMR(400 MHz, CDCl_3_) δ = 7.23-7.27 (m, 1H), 7.11-7.13 (m, 2H), 6.80-6.83 (m, 1H), 5.05 (sep, *J* = 6.4 Hz, 1H), 3.80 (s, 4H), 1.73 (s, 3H), 1.28 (d, *J* = 6.4 Hz, 3H), 1.19 (d, *J* = 6.4 Hz, 3H); ^13^C-NMR (100 MHz, CDCl_3_) δ = 175.1, 159.6, 144.8, 129.3, 117.6, 113.2, 111.0, 75.7, 70.4, 55.3, 26.8, 21.7, 21.5; HRMS *m/z*; calcd. for C_13_H_18_O_4_Na: 261.10973; found 261.10993.

*Isopropyl* *2-hydroxy-2-(3-fluorophenyl)propanoate* (**5aj**): [α]_D_^22^ = +34.50 (c 4.0, CHCl_3_), 90% ee [HPLC conditions: Chiralcel OJ-H column, hexane/2-propanol = 98/2, flow = 1.0 mL/min, wavelength = 230 nm, t_major_ = 5.9 min and t_minor_ = 7.7 min]; ^1^H-NMR (400 MHz, CDCl_3_) δ = 7.25-7.34 (m, 3H), 6.94-6.99 (m, 1H), 5.05 (sep, *J* = 6.4 Hz, 1H), 3.86 (s, 1H), 1.73 (s, 3H), 1.29 (d, *J* = 6.4 Hz, 3H), 1.18 (d, *J* = 6.4 Hz, 3H); ^13^C-NMR (100 MHz, CDCl_3_) δ = 174.8, 162.8 (d, *J* = 245 Hz), 145.7 (d, *J* = 7.63 Hz), 129.8 (d, *J* = 8.58 Hz), 121.0, 114.6 (d, *J* = 20.98 Hz), 112.7 (d, *J* = 23.84 Hz), 75.3, 70.7, 26.8, 21.7, 21.5; HRMS *m/z*; calcd. for C_12_H_15_O_3_FNa: 249.08974; found 249.08997.

*Isopropyl* *2-hydroxy-2-(3-fluoro-4-methoxyphenyl)propanoate* (**5ak**): [α]_D_^22^ = +33.22 (c 5.2, CHCl_3_), 87% ee [HPLC conditions: Chiralcel OJ-H column, hexane/2-propanol = 98/2, flow = 1.0 mL/min, wavelength = 230 nm, t_major_ = 13.1 min and t_minor_ = 21.2 min]; ^1^H-NMR (400 MHz, CDCl_3_) δ = 7.23-7.30 (m, 2H), 6.90 (t, *J* = 8.7 Hz, 1H), 5.03 (sep, *J* = 6.4 Hz, 1H), 3.86 (s, 4H), 1.69 (s, 3H), 1.27 (d, *J* = 6.4 Hz, 3H), 1.17 (d, *J* = 6.4 Hz, 3H); ^13^C-NMR (100 MHz, CDCl_3_) δ = 175.0, 152.0 (d, *J* = 245 Hz), 147.1 (d, *J* = 11.44 Hz), 136.1 (d, *J* = 5.72 Hz), 121.1 (d, *J* = 2.86 Hz), 113.6 (d, *J* = 20 Hz), 112.9, 74.9, 70.6, 56.3, 26.8, 21.7, 21.5; HRMS *m/z*; calcd. for C_13_H_17_O_4_FNa: 279.10031; found 279.10049.

*Isopropyl* *2-hydroxy-2-phenylbutanoate* (**5ba**): [α]_D_^24^ = +38.66 (c 5.2, CHCl_3_), 95% ee [HPLC conditions: Chiralcel OJ-H column, hexane/2-propanol = 98/2, flow = 1.0 mL/min, wavelength = 230 nm, t_major_ = 5.6 min and t_minor_ = 11.0 min]; ^1^H-MR (400 MHz, CDCl_3_) δ = 7.58-7.61 (m, 2H), 7.24-7.36 (m, 3H), 5.06 (sep, *J* = 6.4 Hz, 1H), 3.81 (d, *J* = 0.92 Hz, 1H), 2.16-2.26 (m, 1H), 1.94-2.03 (m, 1H), 1.30 (d, *J* = 6.4 Hz, 3H), 1.19 (d, *J* = 6.4 Hz, 3H), 0.92 (t, *J* =7.3 Hz, 3H); ^13^C-NMR (100 MHz, CDCl_3_) δ = 175.0, 142.2, 128.2, 127.6, 125.6, 78.6, 70.4, 32.8, 21.8, 21.6, 8.1; HRMS *m/z*; calcd. for C_13_H_18_O_3_Na: 245.11482; found 245.11495.

*Isopropyl* *2-hydroxy-2-(4-tolyl)butanoate* (**5bc**): [α]_D_^25^ = +32.62 (c 4.8, CHCl_3_), 91% ee [HPLC conditions: Chiralcel OJ-H column, hexane/2-propanol = 98/2, flow = 1.0 mL/min, wavelength = 230 nm, t_major_ = 6.0 min and t_minor_ = 10.2 min]; ^1^H-NMR (400 MHz, CDCl_3_) δ = 7.47 (d, *J* = 8.2 Hz, 2H), 7.13 (d, *J* = 8.2 Hz, 3H), 5.04 (sep, *J* = 6.4 Hz, 1H), 3.76 (s, 1H), 2.32 (s, 3H), 2.15-2.24 (m, 1H), 1.91-2.00 (m, 1H), 1.29 (d, *J* = 6.4 Hz, 3H), 1.19 (d, *J* = 6.4 Hz, 3H), 0.91 (t, *J* =7.3 Hz, 3H); ^13^C-NMR (100 MHz, CDCl_3_) δ = 175.1, 139.3, 137.2, 128.9, 125.5, 78.5, 70.34, 32.8, 21.8, 21.6, 21.1, 8.1; HRMS *m/z*; calcd. for C_14_H_20_O_3_Na: 259.13047; found 259.13047.

*Isopropyl* *2-hydroxy-2-(4-fluorophenyl)butanoate* (**5bf**): [α]_D_^24^ = +39.18 (c 5.3, CHCl_3_), 93% ee [HPLC conditions: Chiralcel OJ-H column, hexane/2-propanol = 98/2, flow = 1.0 mL/min, wavelength = 230 nm, t_major_ = 5.5 min and t_minor_ = 7.4 min]; ^1^H-NMR (400 MHz, CDCl_3_) δ = 7.55-7.58 (m, 2H), 7.00 (t, *J* = 8.6 Hz, 2H), 5.05 (sep, *J* = 6.4 Hz, 1H), 3.84 (s, 1H), 2.13-2.22 (m, 1H), 1.90-1.99 (m, 1H), 1.30 (d, *J* = 6.4 Hz, 3H), 1.17 (d, *J* = 6.4 Hz, 3H), 0.90 (t, *J* = 7.3 Hz, 3H); ^13^C-NMR (100 MHz, CDCl_3_) δ = 174.80, 162.32 (d, *J* = 245 Hz), 137.8 (d, *J* = 2.86 Hz), 127.5 (d, *J* = 8.58 Hz), 114.9 (d, *J* = 20.98 Hz), 78.2, 70.6, 32.9, 21.8, 21.6, 8.0; HRMS *m/z*; calcd. for C_13_H_17_O_3_FNa: 263.10539; found 263.10540.

*Isopropyl* *2-hydroxy-2-(3-methoxyphenyl)butanoate* (**5bh**): [α]_D_^25^ = +26.66 (c 3.1, CHCl_3_), 91% ee [HPLC conditions: Chiralcel OJ-H column, hexane/2-propanol = 98/2, flow = 1.0 mL/min, wavelength = 230 nm, t_major_ = 8.0 min and t_minor_ = 12.7 min]; ^1^H-NMR (400 MHz, CDCl_3_) δ = 7.16-7.26 (m, 3H), 6.79-6.82 (m, 1H), 5.05 (sep, *J* = 6.4 Hz, 1H), 3.79 (s, 4H), 2.14-2.23 (m, 1H), 1.92-2.01 (m, 1H), 1.30 (d, *J* = 6.4 Hz, 3H), 1.20 (d, *J* = 6.4 Hz, 3H), 0.91 (t, *J* = 7.3 Hz, 3H); ^13^C-NMR (100 MHz, CDCl_3_) δ = 174.8, 159.5, 143.9, 129.1, 118.0, 113.1, 111.3, 78.6, 70.5, 55.3, 32.9, 21.8, 21.61, 8.1; HRMS *m/z*; calcd. for C_12_H_20_O_4_Na: 275.12593; found 275.12485.

*Isopropyl* *2-hydroxy-3-methyl-2-phenylbutanoate* (**5ca**): [α]_D_^22^ = +5.95 (c 3.4, CHCl_3_), 94% ee [HPLC conditions: Chiralcel OJ-H column, hexane/2-propanol = 98/2, flow = 1.0 mL/min, wavelength = 230 nm, t_major_ = 4.1 min and t_minor_ = 5.2 min]; ^1^H-NMR (400 MHz, CDCl_3_) δ = 7.62-7.65 (m, 2H), 7.23-7.34 (m, 3H), 5.03 (sep, *J* = 6.6 Hz, 1H), 3.71 (s, 1H), 2.59 (sep, *J* = 6.9 Hz, 1H), 1.32 (d, *J* = 6.0 Hz, 3H), 1.17 (d, *J* = 6.0 Hz, 3H), 0.98 (d, *J* = 6.8 Hz, 3H), 0.68 (d, *J* = 6.9 Hz, 3H); ^13^C-NMR (100 MHz, CDCl_3_) δ = 175.3, 141.5, 128.0, 127.4, 126.0, 80.7, 70.5, 35.8, 21.8, 21.6, 17.1, 15.9; HRMS *m/z*; calcd. for C_14_H_20_O_3_Na: 259.13047; found 259.13042.

*Isopropyl* *2-hydroxy-3-methyl-2-(4-methoxyphenyl)butanoate* (**5cd**): [α]_D_^22^ = +36.53 (c 3.5, CHCl_3_), 90% ee [HPLC conditions: Chiralcel OJ-H column, hexane/2-propanol = 98/2, flow = 1.0 mL/min, wavelength = 230nm, t_major_ = 5.9 min and t_minor_ = 7.1 min]; ^1^H-NMR (400 MHz, CDCl_3_) δ = 7.54 (d, *J* = 9.1 Hz, 2H), 6.85 (d, *J* = 9.1 Hz, 2H), 5.02 (sep, *J* = 6.4 Hz, 1H), 3.79 (s, 3H), 3.68 (s, 1H), 2.54 (sep,*J* = 6.9 Hz, 1H), 1.31 (d, *J* = 6.4 Hz, 3H), 1.17 (d, *J* = 6.4 Hz, 3H), 0.96 (d, *J* = 6.4 Hz, 3H), 0.68 (d, *J* = 7.3 Hz, 3H); ^13^C-NMR (100 MHz, CDCl_3_) δ = 175.5, 158.9, 133.6, 127.2, 113.3, 80.4, 70.4, 55.3, 35.7, 21.8, 21.6, 17.0, 15.9; HRMS *m/z*; calcd. for C_15_H_22_O_4_Na: 289.14103; found 289.14093.

*Isopropyl* *2-hydroxy-2-phenyl-2-(4-tolyl)acetate* (**5dc**): [α]_D_^24^ = −4.14 (c 5.2, CHCl_3_), 92% ee [HPLC conditions: Chiralcel OJ-H column, hexane/2-propanol = 98/2, flow = 1.0 mL/min, wavelength = 230 nm, t_major_ = 18.4 min and t_minor_ = 20.8 min]; ^1^H-NMR (400 MHz, CDCl_3_) δ = 7.42-7.44 (m, 2H), 7.29-7.34 (m, 5H), 7.13 (d, *J* = 8.2 Hz, 2H), 5.14 (sep, *J* = 6.4 Hz, 1H), 4.26 (s, 1H), 2.34 (s, 3H), 1.24 (t, *J* = 6.4 Hz, 6H); ^13^C-NMR (100 MHz, CDCl_3_) δ = 174.2, 142.3, 139.3, 137.7, 128.8, 128.0, 127.9, 127.5, 127.4, 80.7, 71.2, 21.6, 21.2; HRMS *m/z*; calcd. for C_18_H_20_O_3_Na: 307.13047; found 307.13070.

*Isopropyl 2--2-hydroxy-2-(4-methoxyphenyl)-2-phenylacetate* (**5dd**): [α]_D_^25^ = +2.10 (c 4.0, CHCl_3_), 86% ee [HPLC conditions: Chiralcel AS-H column, hexane/2-propanol = 9/1, flow = 1.0 mL/min, wavelength = 230 nm, t_major_ = 20.2 min and t_minor_ = 22.8 min]; ^1^H-NMR (400 MHz, CDCl_3_) δ = 7.43-7.45 (m, 2H), 7.30-7.36 (m, 5H), 6.87-6.85 (d, *J* = 8.7 Hz, 2H), 5.15 (sep, *J* = 6.4 Hz, 1H), 4.28 (d, *J* = 2.3 Hz, 1H), 3.80 (s, 3H), 1.24 (dd, *J* = 6.4, 6.4Hz, 6H); ^13^C-NMR (100 MHz, CDCl_3_) δ = 174.2, 159.3, 142.4, 134.4, 128.8, 128.1, 128.0, 127.5, 113.4, 80.5, 71.2, 55.4, 21.6, 21.6; HRMS *m/z*; calcd. for C_18_H_20_O_4_Na: 323.12593; found 323.12614.

*Isopropyl 2-(4-chlorophenyl)-2-hydroxy-2-phenylacetate* (**5de**) [47]: [α]_D_^20^ = +17.87 (c 4.9, CHCl_3_), 91% ee [HPLC conditions: Chiralcel AD-H column, hexane/2-propanol = 9/1, flow = 1.0 mL/min, wavelength = 230 nm, t_minor_ = 18.9 min and t_major_ = 20.0 min]; ^1^H-NMR (400 MHz, CDCl_3_) δ = 7.28-7.40 (m, 9H), 5.15 (sep, *J* = 6.4 Hz, 1H), 4.30 (s, 1H), 1.24 (dd, *J* = 6.4, 6.0 Hz, 6H); ^13^C-NMR (100 MHz, CDCl_3_) δ = 173.6, 142.0, 140.6, 134.0, 129.0, 128.3, 128.2, 128.2, 127.3, 80.4, 71.5, 21.6; HRMS m/z; calcd. for C_17_H_17_O_3_ClNa: 327.07584; found 327.07589.

*Isopropyl* *2-(4-fluorophenyl)-2-hydroxy-2-phenylacetate* (**5df**): [α]_D_^24^ = +15.96 (c 4.9, CHCl_3_), 94% ee [HPLC conditions: Chiralcel OJ-H column, hexane/2-propanol = 98/2, flow = 1.0 mL/min, wavelength = 230 nm, t_major_ = 10.3 min and t_minor_ = 11.4 min]; ^1^H-NMR (400 MHz, CDCl_3_) δ = 7.30-7.44 (m, 7H), 7.00 (t, *J* = 8.7 Hz, 2H), 5.15 (sep, *J* = 6.4 Hz, 1H), 4.31 (s, 1H), 1.24 (dd, *J* = 6.4, 6.4 Hz, 6H); ^13^C-NMR (100 MHz, CDCl_3_) δ = 173.9, 162.5 (d, *J* = 247.0 Hz), 142.1, 137.9 (d, *J* = 2.86 Hz), 129.40 (d, *J* = 8.58 Hz), 128.23, 128.17, 127.3, 114.9 (d, *J* = 20.98 Hz), 80.4, 71.4, 21.6; HRMS *m/z*; calcd. for C_17_H_17_O_3_FNa: 311.10539; found 311.10545.

*Isopropyl* *2-hydroxy-2-(3-methoxyphenyl)-2-phenylacetate* (**5dh**): [α]_D_^25^ = −3.95 (c 2.7, CHCl_3_), 92% ee [HPLC conditions: Chiralcel AD-H column, hexane/2-propanol = 9/1, flow = 0.85 mL/min, wavelength = 230 nm, t_major_ = 14.5 min and t_minor_ = 15.3 min]; ^1^H-NMR (400 MHz, CDCl_3_) δ = 7.41-7.44 (m, 2H), 7.22-7.35 (m, 4H), 7.01-7.03 (m, 2H), 6.84-7.03 (m, 1H), 5.15 (sep, *J* = 6.4 Hz, 1H), 4.32 (s, 1H), 3.76 (s, 3H), 1.25 (t, *J* = 6.5 Hz, 6H); ^13^C-NMR (100 MHz, CDCl_3_) δ = 173.9, 159.4, 143.6, 142.0, 129.1, 128.1, 128.0, 127.5, 120.0, 113.6, 113.2, 80.8, 71.3, 55.3, 21.6, 21.6; HRMS *m/z*; calcd. for C_18_H_20_O_4_Na: 323.12593; found 323.12638.

*Isopropyl 2-(3-chlorophenyl)-2-(4-fluorophenyl)-2-hydroxyacetate* (**5ei**): [α]_D_^22^ = +1.23 (c 5.3, CHCl_3_), 90% ee [HPLC conditions: Chiralcel OJ-H column, hexane/2-propanol = 98/2, flow = 1.0 mL/min, wavelength = 230 nm, t_major_ = 10.9 min and t_minor_ = 12.2 min]; ^1^H-NMR (400 MHz, CDCl_3_) δ = 7.25-7.45 (m, 6H), 7.02 (m, 2H), 5.16 (sep, *J* = 6.4 Hz, 1H), 1.25 (d, *J* = 6.4 Hz, 6H); ^13^C-NMR (100 MHz, CDCl_3_) δ = 173.3, 162.6 (d,*J* = 247 Hz), 143.9, 137.4 (d, *J* = 2.86 Hz), 134.2, 129.4, 129.2 (d, *J* = 8.58 Hz), 128.4, 127.6, 125.7, 115.1 (d, *J* = 20.98 Hz), 79.9, 71.8, 21.6; HRMS *m/z*; calcd. for C_17_H_16_O_3_ClFNa: 345.06642; found 345.06639.

## 4. Conclusions

In summary, we have developed a catalytic asymmetric arylation of aliphatic aldehydes and α-ketoesters with arylboronic acids by RuCl_2_(p-cymene)/Me-BIPAM catalyst. With this catalyst system, a broad range of enantiopure alkyl(aryl)methanols and α-hydroxy-esters were easily prepared. Studies on further applications of Me-BIPAM to other C-C bond-forming reactions are in progress in our group. 
